# Traumatized refugees: identifying needs and facing challenges for mental health care

**DOI:** 10.1080/20008198.2017.1388103

**Published:** 2017-11-07

**Authors:** Christine Knaevelsrud, Nadine Stammel, Miranda Olff

**Affiliations:** ^a^ European Journal of Psychotraumatology, Department of Education and Psychology, Division of Clinical Psychological Intervention, Freie Universität Berlin, Germany; ^b^ Center Überleben (former Center for Torture Victims), Berlin, Germany; ^c^ European Journal of Psychotraumatology, Department of Psychiatry, Academic Medical Center, University of Amsterdam; ^d^ Arq Psychotrauma Expert Group, Diemen, The Netherlands

**Keywords:** refugees, trauma, PTSD, mental health, screening, intervention

## Abstract

In the past few years the number of refugees worldwide has increased dramatically. Many of them were traumatized in their homelands due to violent conflict or persecution, as well as during their flight, and are confronted with ongoing stressors in the exile countries. In order to contribute to enhancing the clinical knowledge, this special issue of the *European Journal of Psychotraumatology* focuses on traumatized refugees. It includes three review articles as well as four original articles on the mental health burden, screening instruments and interventions in different groups of refugees. The articles published in this special issue focus on important aspects of better understanding the needs of traumatized refugees, as well as on identifying and offering appropriate mental health care for this population. Future research recommendations are provided in the hope to contribute to improving mental health care strategies of this still underserved population.

International studies show that refugees suffer from increased rates of short-term and long-term mental health problems: approximately one in three refugees is suffering from PTSD or other trauma-related mental disorder (Bogic, Njoku, & Priebe, 2015; Fazel, Wheeler, & Danesh, 2005; Steel et al., 2009). Despite the urgency to offer treatment, there is limited knowledge about the mental health burden of, and effective interventions for, traumatized refugees (Acarturk et al., 2015; ter Heide, Mooren, & Kleber, 2016). Currently, the best evidence for reducing trauma-related mental health problems in refugees can be found for trauma-focused interventions, such as narrative exposure therapy (Crumlish & O’Rourke, 2010; Nickerson, Bryant, Silove, & Steel, 2011; Nosè et al., 2017). Other forms of psychosocial interventions are not well studied. There is a need to study psychosocial interventions in different refugee populations, focusing on their effectiveness but also on aspects of feasibility and cultural adaptation.

*European Journal of Psychotraumatology* has a strong tradition focusing on global mental health issues and populations forced to migrate (see special issues Hall & Olff, 2016; Purgato & Olff, 2015; Turner, 2015). To enhance clinical knowledge, this special issue focuses on traumatized refugees. It includes three review articles as well as four original articles on the mental health burden, screening instruments and interventions in different groups of refugees. The first article gives an overview on the mental health status in North Korean refugees in South Korea and focuses on the risk and protective factors in this little studied population (Lee, Park, & Lee, 2017). The first review ever conducted on the mental health of North Korean refugees summarizes the findings of 56 studies, reporting high prevalence rates of symptoms of posttraumatic stress disorder (PTSD), depression and anxiety. Risk and protective factors, pre- and post-settlement factors as well as personal factors associated with mental health are presented. The authors identify several risk factors, such as traumatic exposure in North Korea and during flight, acculturative stress, poor physical health and age. Repatriation and length of stay in a third country seem to be additional risk factors specific for this population; protective factors include higher educational and economic background before flight and social support.

In a systematic review and meta-analysis, an overview of the effectiveness of psychosocial interventions for displaced war-traumatized minors is provided focusing on symptoms of PTSD, depression, anxiety, grief and general distress (Nocon, Eberle-Sejari, Unterhitzensberger, & Rosner, 2017). The authors conclude that Cognitive Behavioural therapy (CBT) and Interpersonal Therapy showed promising results, needing further replication. Few treatment studies were available for the population of children and adolescents, mostly with low methodological quality, and most of the interventions resulted in no significant improvements of mental health problems.

The third review article focuses on the special situation of Syrian refugees, their mental health conditions, and scalable evidence-based interventions. It outlines the newly launched STRENGTHS programme for adapting, scaling up and testing Problem Management interventions (Sijbrandij et al., 2017).

Very little research has been done on the validity of instruments for refugee populations (Wind, van der Aa, de la Rie, & Knipscheer, 2017). The first research article in this special issue describes the evaluation of the Refugee Health Screener 15 (RHS-15), a screening instrument for common mental health problems in refugees, in a sample of 86 refugees residing in Germany (Kaltenbach, Härdtner, Hermenau, Schauer, & Elbert, 2017). The RHS-15, as well as the shorter RHS-13, showed good feasibility, reliability and validity in the self-rating as well as in the interview version.

This issue continues with the cultural adaptation and evaluation of a CBT programme for Farsi-speaking refugees, that was conducted with male refugees from Afghanistan and Iran (Kananian, Ayoughi, Farugie, Hinton, & Stangier, 2017). The transdiagnostic intervention conducted in a group setting shows promising results in terms of reducing general psychopathological stress and improving quality of life.

In the third research article, 76 adult patients at a treatment centre for torture victims and traumatized refugees were surveyed in the context of their regular multidisciplinary psychotherapeutic treatment (Stammel et al., 2017). The study shows that symptoms of trauma-associated disorders decline and quality of life improves over the course of treatment. Younger age was associated with greater reductions of somatoform symptoms.

Finally, a group-based multidisciplinary day patient treatment for refugees who experienced multiple traumatic losses was evaluated (de Heus et al., 2017). In this article, data from 14 patients who participated was analysed, supporting the feasibility and potential effectiveness of the programme.

The articles published in this special issue focus on important aspects to better understand the needs of traumatized refugees, as well as on identifying and offering appropriate mental health care for this population. Cultural sensitive psychotraumatology remains a priority (Schnyder et al., 2016) and rigorous treatment research, whether randomized controlled trials or innovative intervention research paradigms, is needed. For future research the need for low-threshold, cost-effective interventions for refugees with mental health problems, such as online-supported interventions or transdiagnostic approaches, should be considered and alternative types of interventions – integrative and complementary approaches – as well as making better use of e-health opportunities deserve further investigation (see call for papers). The *European Journal of Psychotraumatology* looks forward to receiving more papers focusing on traumatized refugees, to improve the mental health care strategies of this underserved population.

## References

[CIT0001] Acarturk C., Konuk E., Cetinkaya M., Senay I., Sijbrandij M., Cuijpers P., & Aker T. (2015). EMDR for Syrian refugees with posttraumatic stress disorder symptoms: Results of a pilot randomized controlled trial. *European Journal of Psychotraumatology*, 6(1), 27414. doi:10.3402/ejpt.v6.27414 25989952 PMC4438099

[CIT0002] Bogic M., Njoku A., & Priebe S. (2015). Long-term mental health of war-refugees: A systematic literature review. *BMC International Health and Human Rights*, 15, 29. doi:10.1186/s12914-015-0064-9 26510473 PMC4624599

[CIT0003] Crumlish N., & O’Rourke K. (2010). A systematic review of treatments for post-traumatic stress disorder among refugees and asylum-seekers. *The Journal of Nervous and Mental Disease*, 198(4), 237–3. doi:10.1097/NMD.0b013e3181d61258 20386252

[CIT0004] de Heus A., Hengst S. M. C., de la Rie S. M., Djelantik M. J., Boelen P. A., & Smid G. E. (2017). Day patient treatment for traumatic grief: Preliminary evaluation of a one-year treatment programme for patients with multiple and traumatic losses. *European Journal of Psychotraumatology*, 8, doi:10.1080/20008198.2017.1375335 PMC5632766 29038679

[CIT0005] Fazel M., Wheeler J., & Danesh J. (2005). Prevalence of serious mental disorder in 7000 refugees resettled in western countries: A systematic review. *Lancet*, 365(9467), 1309–1314. S0140-6736(05)61027-6 [pii]. doi:10.1016/S0140-6736(05)61027-6 15823380

[CIT0006] Hall B. J., & Olff M. (2016). Global mental health: Trauma and adversity among populations in transition. *European Journal of Psychotraumatology*, 7, 31140. doi:10.3402/ejpt.v7.31140 PMC4756626 26886490

[CIT0007] Kaltenbach E., Härdtner E., Hermenau K., Schauer M., & Elbert T. (2017). Efficient identification of mental health problems in refugees in Germany - The Refugee Health Screener. *European Journal of Psychotraumatology, 8*(S2), 1389205. doi:10.1080/20008198.2017.1389205 PMC5687797 29163869

[CIT0008] Kananian S., Ayoughi S., Farugie A., Hinton D., & Stangier U. (2017). Transdiagnostic culturally adapted CBT with Farsi-speaking refugees: A pilot study. *European Journal of Psychotraumatology, 8*(S2), 1390362. doi:10.1080/20008198.2017.1390362 PMC5687805 29163870

[CIT0009] Lee Y., Park S., & Lee M. (2017). Mental health status of North Korean refugees in South Korea and risk and protective factors: a 10-year review of the literature. *European Journal of Psychotraumatology, 8*(S2), 1369833. doi:10.1080/20008198.2017.1369833 PMC5632770 29038687

[CIT0010] Nickerson A., Bryant R. A., Silove D., & Steel Z. (2011). A critical review of psychological treatments of posttraumatic stress disorder in refugees. *Clinical Psychology Review*, 31(3), 399–417. S0272-7358(10)00170-4[pii]. doi:10.1016/j.cpr.2010.10.004 21112681

[CIT0011] Nocon A., Eberle-Sejari R., Unterhitzensberger J., & Rosner R. (2017). The Effectiveness of psychosocial Interventions in war-traumatized refugee and internally displaced Minors - Systematic Review and Meta-analysis. *European Journal of Psychotraumatology, 8*(S2), 1388709. doi:10.1080/20008198.2017.1388709 PMC5687794 29163868

[CIT0012] Nosè M., Ballette F., Bighelli I., Turrini G., Purgato M., Tol W., … Schmahl C. (2017). Psychosocial interventions for post-traumatic stress disorder in refugees and asylum seekers resettled in high-income countries: Systematic review and meta-analysis. *PLoS One*, 12(2), e0171030. doi:10.1371/journal.pone.0171030 28151992 PMC5289495

[CIT0013] Purgato M., & Olff M. (2015). Global mental health and trauma: The current evidence and the long road ahead. *European Journal of Psychotraumatology*, 6, 1. doi:10.3402/ejpt.v6.30120 PMC4654766 26589260

[CIT0014] Schnyder U., Bryant R. A., Ehlers A., Foa E. B., Hasan A., Mwiti G., … Yule W. (2016). Culture-sensitive psychotraumatology. *European Journal of Psychotraumatology*, 7(1), 31179. doi:10.3402/ejpt.v7.31179 27473520 PMC5055610

[CIT0015] Sijbrandij M., Acarturk C., Bird M., Bryant R. A., Burchert S., Carswell K., … Cuijpers P. (2017). Strengthening mental health care systems for Syrian refugees in Europe and the Middle East: Integrating scalable psychological interventions in 8 countries. *European Journal of Psychotraumatology*, *8*(S2), 1388102. doi:10.1080/20008198.2017.1388102 PMC5687806 29163867

[CIT0016] Stammel N., Knaevelsrud C., Schock K., Walther L., Wenk-Ansohn M., & Böttche M. (2017). Multidisciplinary treatment for traumatized refugees in a naturalistic setting: Symptom courses and predictors. *European Journal of Psychotraumatology*, *8*(S2), 1377552. doi:10.1080/20008198.2017.1377552 PMC5687793 29163866

[CIT0017] Steel Z., Chey T., Silove D., Marnane C., Bryant R. A., & van Ommeren M. (2009). Association of torture and other potentially traumatic events with mental health outcomes among populations exposed to mass conflict and displacement: A systematic review and meta-analysis. *JAMA*, 302(5), 537–549. 302/5/537 [pii]. doi:10.1001/jama.2009.1132 19654388

[CIT0018] ter Heide F. J. J., Mooren T. M., & Kleber R. J. (2016). Complex PTSD and phased treatment in refugees: A debate piece. *European Journal of Psychotraumatology*, 7(1), 28687. doi:10.3402/ejpt.v7.28687 26886486 PMC4756628

[CIT0019] Turner S. (2015). Refugee blues: A UK and European perspective. *European Journal of Psychotraumatology*, 6, 29328. doi:10.3402/ejpt.v6.29328 PMC4626650 26514159

[CIT0020] United Nations High Commissioner for Refugees (UNHCR) (2017). *Global trends: Forced displacement in 2016*. UNHCR: Geneva.

[CIT0021] Wind T. R., van der Aa N., de la R. S., & Knipscheer J. (2017). The assessment of psychopathology among traumatized refugees: Measurement invariance of the Harvard Trauma Questionnaire and the Hopkins Symptom Checklist-25 across five linguistic groups. *European Journal of Psychotraumatology*, 8(S2), 1321357. doi:10.1080/20008198.2017.1321357 29038686 PMC5632793

